# P-503. Perspectives of Community Health Workers on the Rise of Congenital Syphilis

**DOI:** 10.1093/ofid/ofaf695.718

**Published:** 2026-01-11

**Authors:** John M Flores, Nikki Kasal, Alicia Dawdani, Caroline Montag, Declan Quinn, Josie Majowka, Kaitlyn Gomez, Lilly Cheng-Immergluck, John Schneider

**Affiliations:** Cook County Health / University of Chicago, Chicago, IL; University of Chicago Pritzker School of Medicine, Chicago, Illinois; University of Chicago, Chicago, Illinois; University of Chicago Pritzker School of Medicine, Chicago, Illinois; University of Chicago, Chicago, Illinois; University of Chicago Medicine, Chicago, Illinois; University of Chicago, Chicago, Illinois; University of Chicago, Chicago, Illinois; University of Chicago, Chicago, Illinois

## Abstract

**Background:**

Congenital syphilis (CS) incidence has been increasing for the past decade. Previous studies have identified various barriers associated with the entry, retention and linkage into care along the CS Prevention Cascade. In order to develop a targeted intervention to engage community health workers (CHWs) on the CS Prevention Cascade, we conducted a qualitative study to gather their perspective on factors associated with the rise of CS.

**Figure 1:**
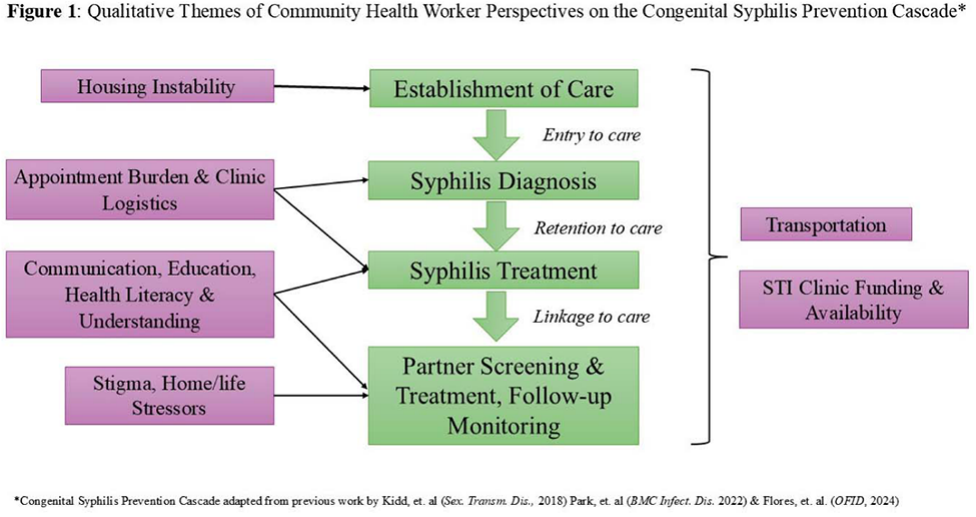
Qualitative Themes of Community Health Worker Perspectives on the Congenital Syphilis Prevention Cascade*

**Table 1:**
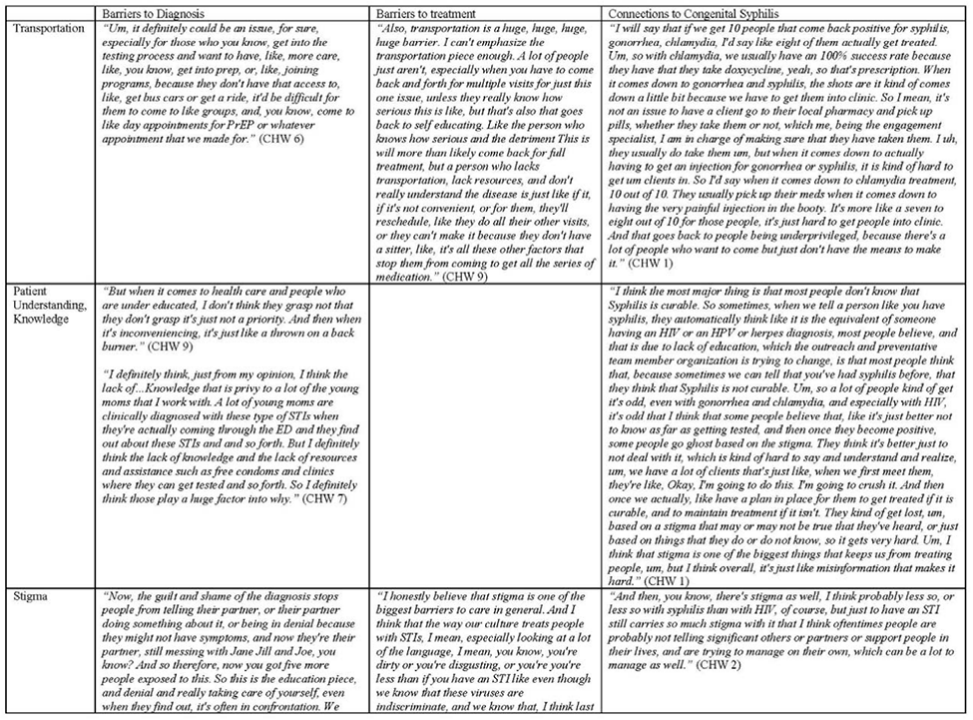
Perceived barriers to care leading to acquisition of syphilis and other sexually transmitted infection among women of childbearing age, from the perspective of community health workers.

**Methods:**

From Chicago, IL, USA, we recruited CHWs who work directly with women of childbearing age in health systems with high CS incidence, to participate in semi-structured interviews. Questions were related to prenatal and obstetric care, syphilis clinical care, sociostructural and psychosocial factors related to the diagnosis. Thematic qualitative analyses were performed to analyze perceived barriers to STI care and management, including maternal syphilis.

**Table 1:**
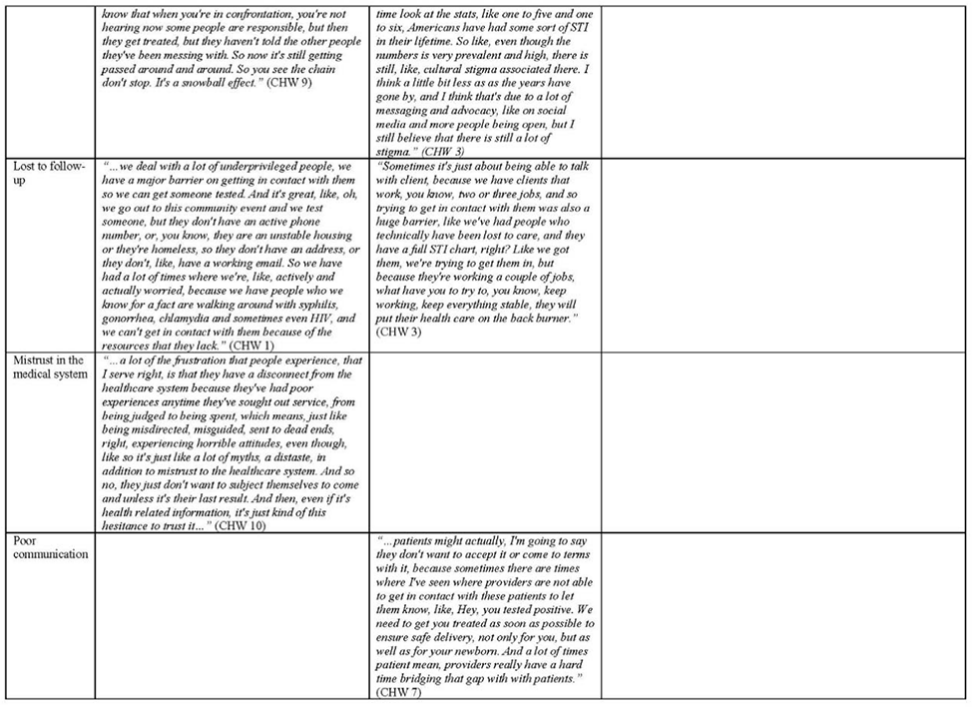
Perceived barriers to care leading to acquisition of syphilis and other sexually transmitted infection among women of childbearing age, from the perspective of community health workers.

**Results:**

12 CHWs from 5 different organizations completed the study. Generally, the most frequently cited barriers to syphilis care for women of childbearing age are transportation and patient understanding/knowledge, followed by stigma, lost to clinical follow-up, mistrust in the medical system and poor communication. Additional factors identified included issues related to appointment burden, financial stressors, domestic partner violence, inadequate partner testing and treatment, disclosure to partner, substance and alcohol use, and mental health comorbidities. The CHWs all described interest in engaging patients to help overcome these factors including assisting in transportation, education initiatives, and improved communication cohesion with providers. (Figure 1, Table 1)

**Conclusion:**

Numerous factors identified by CHWs were associated with inadequate testing and treatment of syphilis among women of childbearing age. This data is pivotal to creating novel and effective interventions that engage CHWs along the CS Prevention Cascade, and contribute to reducing the burden of this preventable disease.

**Disclosures:**

Lilly Cheng-Immergluck, MD, MS, American Academy of Pediatrics: Board Member|Department of Energy: Grant/Research Support|National Institutes of Health: Grant/Research Support|Pfizer: Grant/Research Support|Sanofi: Grant/Research Support

